# Latitudinal Gradients in Degradation of Marine Dissolved Organic Carbon

**DOI:** 10.1371/journal.pone.0028900

**Published:** 2011-12-28

**Authors:** Carol Arnosti, Andrew D. Steen, Kai Ziervogel, Sherif Ghobrial, Wade H. Jeffrey

**Affiliations:** 1 Department of Marine Sciences, University of North Carolina, Chapel Hill, North Carolina, United States of America; 2 Center for Environmental Diagnostics and Bioremediation, University of West Florida, Pensacola, Florida, United States of America; Northeastern University, United States of America

## Abstract

Heterotrophic microbial communities cycle nearly half of net primary productivity in the ocean, and play a particularly important role in transformations of dissolved organic carbon (DOC). The specific means by which these communities mediate the transformations of organic carbon are largely unknown, since the vast majority of marine bacteria have not been isolated in culture, and most measurements of DOC degradation rates have focused on uptake and metabolism of either bulk DOC or of simple model compounds (e.g. specific amino acids or sugars). Genomic investigations provide information about the potential capabilities of organisms and communities but not the extent to which such potential is expressed. We tested directly the capabilities of heterotrophic microbial communities in surface ocean waters at 32 stations spanning latitudes from 76°S to 79°N to hydrolyze a range of high molecular weight organic substrates and thereby initiate organic matter degradation. These data demonstrate the existence of a latitudinal gradient in the range of complex substrates available to heterotrophic microbial communities, paralleling the global gradient in bacterial species richness. As changing climate increasingly affects the marine environment, changes in the spectrum of substrates accessible by microbial communities may lead to shifts in the location and rate at which marine DOC is respired. Since the inventory of DOC in the ocean is comparable in magnitude to the atmospheric CO_2_ reservoir, such a change could profoundly affect the global carbon cycle.

## Introduction

Marine DOC (dissolved organic carbon) is one of the largest actively cycling reservoirs of organic carbon on earth, comparable in magnitude to the atmospheric reservoir of CO_2_ [1]; heterotrophic microbial communities play a key role in driving the DOC cycle [2]–[4]. DOC consists of many thousands of different compounds, and is operationally divided into labile, semi-labile, and recalcitrant fractions that are defined based on timescales of removal in bioassays or by direct measurement in the ocean [5]. Structural or mechanistic explanations for varying timescales of DOC degradation, however, are lacking [6]. Since heterotrophic bacteria are unable to transport directly into the cell most substrates with a molecular weight greater than 600 Da [7], hydrolysis via extracellular enzymes is required prior to substrate uptake. The requirement for enzymatic hydrolysis is therefore a promising starting point to search for mechanistic explanations of variations in the abilities of marine bacteria to utilize specific fractions of DOC as a substrate. The activities and structural specificities of polysaccharide-hydrolyzing enzymes are of particular importance in this respect, since carbohydrates constitute a large proportion of marine high molecular weight DOC: 54% of surface water DOC and 25% of DOC in the deep ocean [8]. Most of the rest of DOC is classified as ‘uncharacterized’ on a molecular basis, since lipids, amino acids, and amino sugars together constitute less than ca. 5% of the total [9].

Assessing the enzymatic capabilities of marine heterotrophic microbial communities can best be done directly in seawater, since cultured microbial isolates constitute only a small, unrepresentative fraction of extent marine microbes [10]. Most measurements of enzyme activity in seawater are based on small chromogenic or fluorogenic substrates (e.g. [11]), which provide very little information about enzymatic substrate specificities [12]. Genomic investigations can yield valuable insights into community and organism potential [13]–[15], but provide no information about the extent to which such potential might be expressed. Metatranscriptomic profiling is a promising route to investigate the extent to which genetic potential is realized, but assignment of sequences from the environment to functions such as specific enzyme activities is still problematic due to limitations in database coverage [16] and the vast structural and functional diversity of polysaccharide-hydrolyzing enzymes [17].

To gain insight into the capabilities of natural microbial communities to access polysaccharides, we measured extracellular enzyme activities in surface waters at 32 stations in the Atlantic, Pacific, Arctic, and Southern Oceans, as well as the Gulf of Mexico, spanning latitudes from 76°S to 79°N and a temperature range from −1.8°C to 29°C (Table 1; Fig. S1). We focused on direct detection of the hydrolysis of specific polysaccharides, rather than on investigations of genetic potential, in order to measure the abilities of microbial communities to access substrates irrespective of the multiplicity of enzyme(s) [18] —of known and unknown sequence—that might hydrolyze a specific substrate. Since determining the specific structures of marine dissolved carbohydrates is not possible using currently available analytical techniques [19], we used as substrates a suite of polysaccharides that are components of marine algae [20], and/or whose hydrolytic enzymes have been identified in the genomes of recently-sequenced marine bacteria [13], [15], [21]. These polysaccharides—laminarin, xylan, fucoidan, arabinogalactan, pullulan, and chondroitin sulfate—are structurally diverse, vary in monomer composition and linkage position, and because they are constituents of marine plankton, many are present in considerable quantities in the ocean. The production of laminarin by diatoms and *Phaeocystis* in the ocean, for example, has been estimated at 5 to15 billion metric tons annually [22]. A previous investigation at a few locations had shown evidence of spatial variations in microbial extracellular enzyme activities in surface ocean waters [23]. The present study, the culmination of a series of investigations carried out over the course of a decade, demonstrates that there are recognizable patterns in microbial potential to access DOC on large-scale gradients, mirroring emerging patterns of microbial biogeography in the ocean [24]–[28].

**Table 1:** Station locations and water temperatures.

Station	Latitude	Longitude	Water temp. (°C)
J	79.4N	11.1E	4
AB	77.4N	15.1E	4
P2	66.5N	168.1W	8.7
DO	38.4N	74.6W	13.5
CO	36.4N	74.8W	22
P10	35.5N	164.2W	24.2
GOM1	30.2N	87.4W	28
GOM11	29.6N	87W	28
GOM072	28.5N	89.4W	28.7
GOM073	28.3N	89.4W	28.9
P15	15.5N	161.4W	27.4
BOT12	15.1N	105.5W	29.1
BOT10	10.2N	99.4W	28.6
BOT8	5.4N	92.4W	27.4
T33	1.1N	83.5W	26
BOT7	0.005S	86W	24.1
P21	7.2S	168.4W	29.2
BOT5	8.2S	83.5W	19.8
BOT4	12.2S	81.4W	18
BOT3	17S	79.1W	17.3
T15	23.1S	79.2W	17.5
BOT1	26.3S	75W	15.2
P27	26.5S	173W	21.1
T3	39.2S	77.6W	11
G1	49.3S	174.4W	8.5
R1	56.3S	176.2E	6
R3	62.3S	178.4W	−1.5
M9	65S	176W	−1.7
G11B	74.3S	173.3W	0
R10C	76.1S	170.3E	−1.8
G9A	76.3S	179W	0
R13f	76.5S	177.5E	−1.8

## Results and Discussion

**Figure 1:**
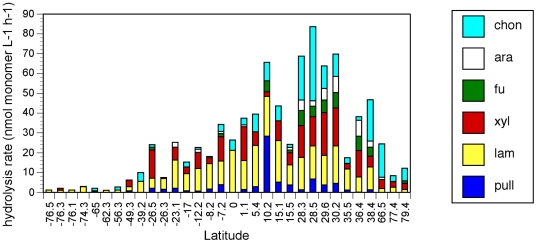
Summed enzymatic hydrolysis rates in surface water at each station plotted against latitude (south latitudes shown with negative numbers). Bar height shows the sum of the maximum enzymatic hydrolysis rate of each substrate at each station. All stations were visited once, with the exception of Station J (79°N); values shown for Station J are averages from 4 visits. (See Fig. 4 and Table S1 for data from each sampling time at Station J.) Pullulan hydrolysis is shown in blue, laminarin in yellow, xylan in red, fucoidan in green, arabinoglactan in white, and chondroitin sulfate in aqua. Hydrolysis rates and standard deviations for all substrates and stations are in Table S1.

**Figure 2:**
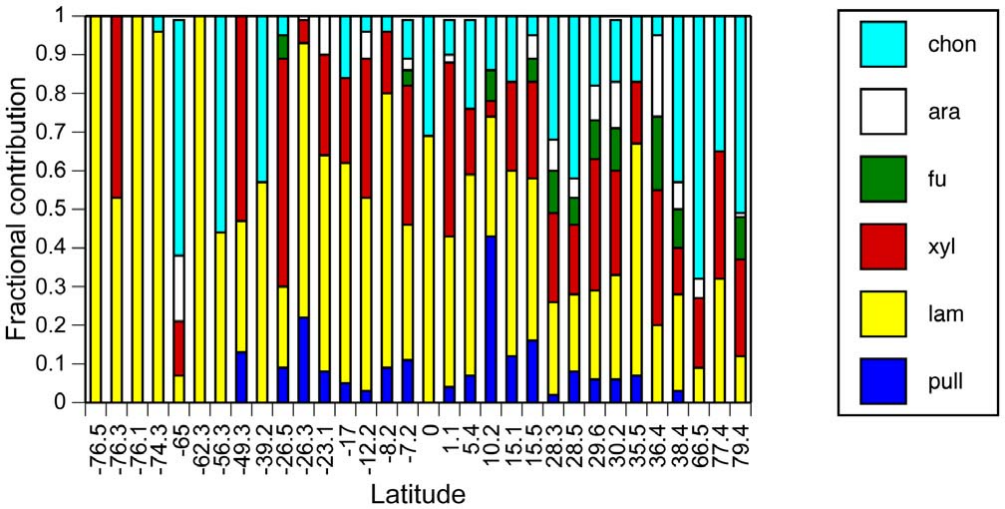
Proportionate contribution of each enzyme activity to summed hydrolysis rates, normalized to 100%. Station latitude and color key for enzyme identity are as in Fig. 2.

**Figure 3:**
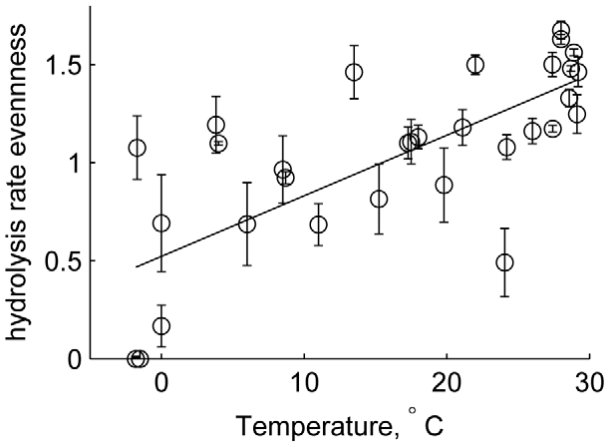
Evenness of enzymatic hydrolysis rates as a function of temperature. Average values (as shown in Figs. 1 and 2) were used for Station J, the only station that was sampled more than once. Line fit: r^2^ = 0.6154, n = 26, p<0.001.

Differing abilities to hydrolyze a diverse range of substrates, as evident in our survey (Fig. 1), demonstrate functional differences among pelagic microbial communities. All substrates were hydrolyzed at only 4 of the 32 stations, all in the Gulf of Mexico. At the other 28 sites, the spectrum of hydrolysis ranged from one to five substrates. Only laminarin was hydrolyzed at every station; chondroitin and xylan were hydrolyzed at the majority (81% and 78%, respectively) of the stations, while pullulan, arabinogalactan, and fucoidan were hydrolyzed at 63%, 47%, and 34% of the stations, respectively. Summed hydrolysis rates were maximal at tropical/subtropical stations, and these summed rates as well as the spectrum of detected enzyme activities, decreased towards the poles (Figs. 1 and 2). The relative contribution of each enzyme activity to the sum at a given station (evenness of hydrolysis rates; Fig. 3) showed a similar pattern, with increasing evenness at higher temperatures (and lower latitudes).

The broad correlation between latitude and summed hydrolysis rates points to the relationship between summed hydrolysis rates and water temperature (n = 35, including 4 visits to a single station (Station J; Table 1), *r^2^* = 0.64, *p*<0.005), a relationship that could be due to the kinetic effect of temperature. This correlation, however, is driven primarily by the correlation with laminarin (*r^2^* = 0.79; *p*<0.005) and by the fact that a broader spectrum of enzyme activities is detected across the range of stations in lower-latitude waters (Figs. 1 and 2). Temperature was poorly correlated with hydrolysis of chondroitin (*r^2^* = 0.15; *p*<0.05), arabinogalactan and pullulan (*r^2^* = 0.20 and 0.21, respectively; *p*<0.01), and fucoidan (*r^2^* = 0.23, p<0.005). The correlation with xylan was stronger (*r^2^* = 0.49, *p*<0.005), but hydrolysis rates of the substrates at stations with similar temperatures varied greatly. At temperatures close to 28°C, for example, the variation in hydrolysis of xylan, fucoidan, arabinogalactan, and chondroitin was an order of magnitude or more (Table 1; Table S1; Fig. 1). Moreover, the differences in hydrolysis rate evenness (Fig. 3) are not explained solely by temperature, since a purely kinetic effect of temperature on hydrolysis rates would be expected to cause a generally proportionate change in the activities of all enzymes, and greatly extended incubation times do not markedly broaden the spectrum of substrates hydrolyzed at high latitude [29].

To the extent that these differences in enzymatic capabilities cannot be explained by temperature, they may derive from variations in microbial community composition that cascade into these functional differences. Arctic microbial communities differ in composition from their temperate counterparts [24], [30]–[31]. Recent investigations have demonstrated latitudinal gradients in microbial community richness, with markedly reduced diversity at high latitudes [26]–[28]. The functional consequences of these variations are unknown, since microbial phylogeny and function are not well correlated. Our results demonstrate that microbial community function varies systematically across latitudinal gradients. The lower summed hydrolysis rates and the more limited spectrum of substrates enzymatically accessible to microbial communities at high latitudes coincides with a reduction in community richness (Figs. 1 and 2).

Differences in the extent to which genes for polysaccharide hydrolases are expressed may also contribute to differences in enzyme activities among microbial communities, but the genetic diversity of polysaccharide hydrolases even among well-studied organisms is barely beginning to be explored [32], complicating efforts for a concerted genetic investigation. One study suggests that a high diversity of hydrolases is potentially available to microbial communities in the North Atlantic [14], but the extent and the conditions under which this genetic potential might be expressed are still matters of speculation. A search of the CAMERA database (http://camera.calit2.net/), for example, yielded numerous gene sequences related to an alpha-l-fucosidase of *Pseudoalteromonas atlantica* T6c (http://img.jgi.doe.gov/cgi-bin/pub/main.cgi). These sequences were found at 60 different Global Ocean Survey (GOS) sites (available through CAMERA), spanning latitudes of 32°S to 45°N, despite the fact that this enzyme activity was not detected in many of our samples from the same range of latitudes (Fig. 1). Likewise, a search (via CAMERA) of the two marine sites in the Antarctic Aquatic Metagenome produced sequences from Newcomb Bay (66°S) closely matching pullulanases from fully-sequenced marine bacteria (http://blast.ncbi.nlm.nih.gov/Blast.cgi), although pullulanase activity was not detectable at any of our sites at latitudes higher than 49°S or 38°N (Fig. 1). Limited geographical overlap between the current CAMERA database and our samples presently preclude a more detailed comparison among sites that would provide insight into the extent to which microbial communities vary in their genetic response to environmental parameters.

**Figure 4:**
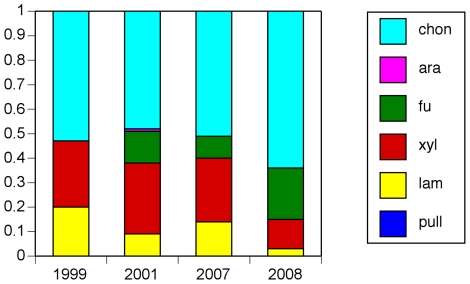
Relative contributions to summed hydrolysis rates for four visits to Station J, Svalbard. Each enzyme activity is shown in a different color; summed rates are normalized to 1.0. Pullulan hydrolysis is shown in blue, laminarin in yellow, xylan in red, fucoidan in green, arabinoglactan in white, and chondroitin sulfate in aqua. All rates plus standard deviations are listed in Table S1.

Patterns of microbial community composition at a given location are temporally repeatable [33]–[37]. Results from our investigation suggest that patterns of hydrolytic activities are also consistent over multi-year timescales. One high-latitude station (79°N, Stn. J; Table 1) sampled four times over the course of 10 years consistently showed hydrolytic activity dominated by chondroitinase (>50% of total activity), with similar patterns and levels of total activity (Table S1; Fig. 4). Likewise, two pairs of stations close to one another in the Gulf of Mexico (GOM1 and GOM11, 28°N; also GOM072 and GOM073, 28.2–30.2°N; Table 1) sampled 6 years apart showed the same broad substrate spectrum (nearly unique among sample locations; Fig. 2) and high levels of total activity (Fig. 1).

The patterns in enzymatic activities observed here provide insight into the potential of microbial communities to access components of the DOC pool, rather than a snapshot of enzyme activities expressed at the time of sampling, since our enzyme incubations lasted several days to two weeks. These experiments demonstrate the potential of a microbial community to access specific substrates over timescales sufficiently long to allow for cellular growth as well as for enzyme induction. The observation that many substrates remained unhydrolyzed over these timescales suggests that some microbial communities collectively lack the capabilities (enzymes, inducers, or organisms) to access specific substrates [38].

This information is unique, since no other currently available analytical method tests the abilities of microbial communities to access specific polysaccharide structures. Likewise, the concentrations of specific polysaccharides in the ocean cannot be measured with currently-available techniques [19], precluding direct measurement of polysaccharide production or concentration. Comparable data cannot be obtained by measuring surface water DOC concentrations at diverse locations, because DOC concentrations are a function of DOC production, DOC degradation, and water mass history [39] (the radiocarbon age of bulk DOC is ca. 6000 years [40].) Measurements of DOC concentrations in the surface ocean therefore cannot differentiate unambiguously between changes in the metabolic capabilities of heterotrophic microbes, changes in DOC production, and differences in water mass history. Moreover, attempts to constrain production and consumption terms for DOC across broad latitudinal gradients are greatly complicated by the multitude of sources and varying bioavailabilities of different components of the DOC pool [6], as well as the paucity of data from high latitude environments [9], [41]. A measure of the spectrum of enzyme activities capable of hydrolyzing a class of biomolecules constituting the largest identified component of the DOC pool thus yields insight into processes otherwise not amenable to quantification.

The broader spectrum of enzyme activities observed at temperate and tropical sites indicates that those microbial communities can access a wider range of substrates than their high-latitude counterparts. The fact that this trend was discernable in our global data set despite variations in season, levels of productivity, oceanic province, and a host of other factors, is remarkable. The functional factors controlling the breadth of microbial community metabolic capabilities remain to be determined. However, the pattern observed here matches the decrease in bacterial species richness observed at high latitudes [26]–[27]. The reason for that trend is not well understood [42]; bacterial richness correlates to a similar extent with water temperature and latitude [26]. A recent model investigation of global patterns of phytoplankton diversity, however, points at the magnitude of seasonal variability in environments and at relative rates of organism dispersal as key factors controlling latitudinal diversity gradients for phytoplankton [43]. Similar factors may control diversity gradients of heterotrophic bacteria.

Projected changes in ocean environments over the coming decades driven by global warming [44]–[45] may increase the rates and widen the spectrum of enzyme activities due to changes in microbial community composition that are facilitated by changes in ocean temperature, circulation, or biogeochemical parameters [46]. Particularly in the Arctic, where rapid temperature increases are projected for the near future, the input of terrestrially-derived DOC into the Arctic basin may be greatly accelerated due to melting of permafrost and increased runoff [47]. Currently, much of this organic matter is buried or exported to the North Atlantic [41], [48]–[49]. In the future, more organic matter may reach the Arctic Ocean, and if the range of complex substrates available to heterotrophic microbial communities is broader in a future Arctic Ocean, a larger fraction of it may be remineralized there.

Kirchman et al. [46] predict that climate change in polar waters may also lead to changes in food web structure resulting in greater transfer of carbon from phytoplankton to DOC to bacteria, and thus to the respiration of a larger fraction of marine primary production to CO_2_. These processes would be greatly facilitated if future polar microbial communities can use a wider range of enzymatic tools, as their temperate to tropical counterparts currently do. Since DOC reactivity is a function of the metabolic capabilities of microbial communities as well as intrinsic chemical characteristics, predictive understanding of the global carbon cycle will require further investigation of the ways in which these metabolic capabilities are likely to change in response to changing climate.

## Materials and Methods

### Sample collection, substrate incubation, and sample analysis

Surface water was collected at each site (Table 1; Fig. S1), by Niskin or Go-Flo bottles; surface water at Stns. J and AB was collected by bucket. Water samples were dispensed into replicate vials. Each vial received a single fluorescently labeled polysaccharide [50] as a substrate, at a concentration of 3.5 µmol monomer equivalent L^−1^. Triplicate vials were incubated at *in situ* temperature. Controls were autoclaved or poisoned with mercuric chloride before substrate addition. Subsamples from each vial were filtered through 0.2 µm pore-sized filters, and were stored frozen prior to analysis. Changes in substrate molecular weight with time were quantified using gel permeation chromatography, with Sephadex G-50 and G-75 columns linked in series, and fluorescence detection (excitation and emission wavelengths of 490 and 530 nm, respectively), as previously described [51]. Hydrolysis rates were calculated from changes in molecular weight distribution of the polysaccharides, as described previously [50]–[51]. No specific permits were required for the collection of water from the ocean as described above. The field studies did not involve any endangered or protected species.

### Statistical analyses

Statistical comparisons were carried out using two-tailed Student's t-test. The evenness of hydrolysis rates (the contribution of each activity to summed activities) was calculated using Shannon's entropy [52], 
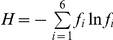
, where *f_i_* represents the hydrolysis rate of the *i*th substrate expressed relative to the sum of all hydrolysis rates for that station. As previously discussed [53], *H* is maximized (*H*≈1.79) when enzymatic hydrolysis rates of all substrates are equal, and minimized (*H* = 0) when only one hydrolysis rate was measurable. Error bars in Fig. 3 represent the standard deviation of the ensemble results from a Monte Carlo error simulation, as described in [53].

## Supporting Information

Figure S1 **Map of sampling locations.**
(TIFF)

Table S1 **Sampling dates and rates of enzymatic hydrolysis (nmol monomer L^−1^ h^−1^) of all substrates at all stations, including standard deviations of triplicate incubations.**
(DOC)
