# Supplementary figures and images for: Latitudinal Gradients in Degradation of Marine Dissolved Organic Carbon
Source: PLoS One. 2011 Dec 28;6(12):e28900. doi: 10.1371/journal.pone.0028900 (PMC3247214; doi:10.1371/journal.pone.0028900)

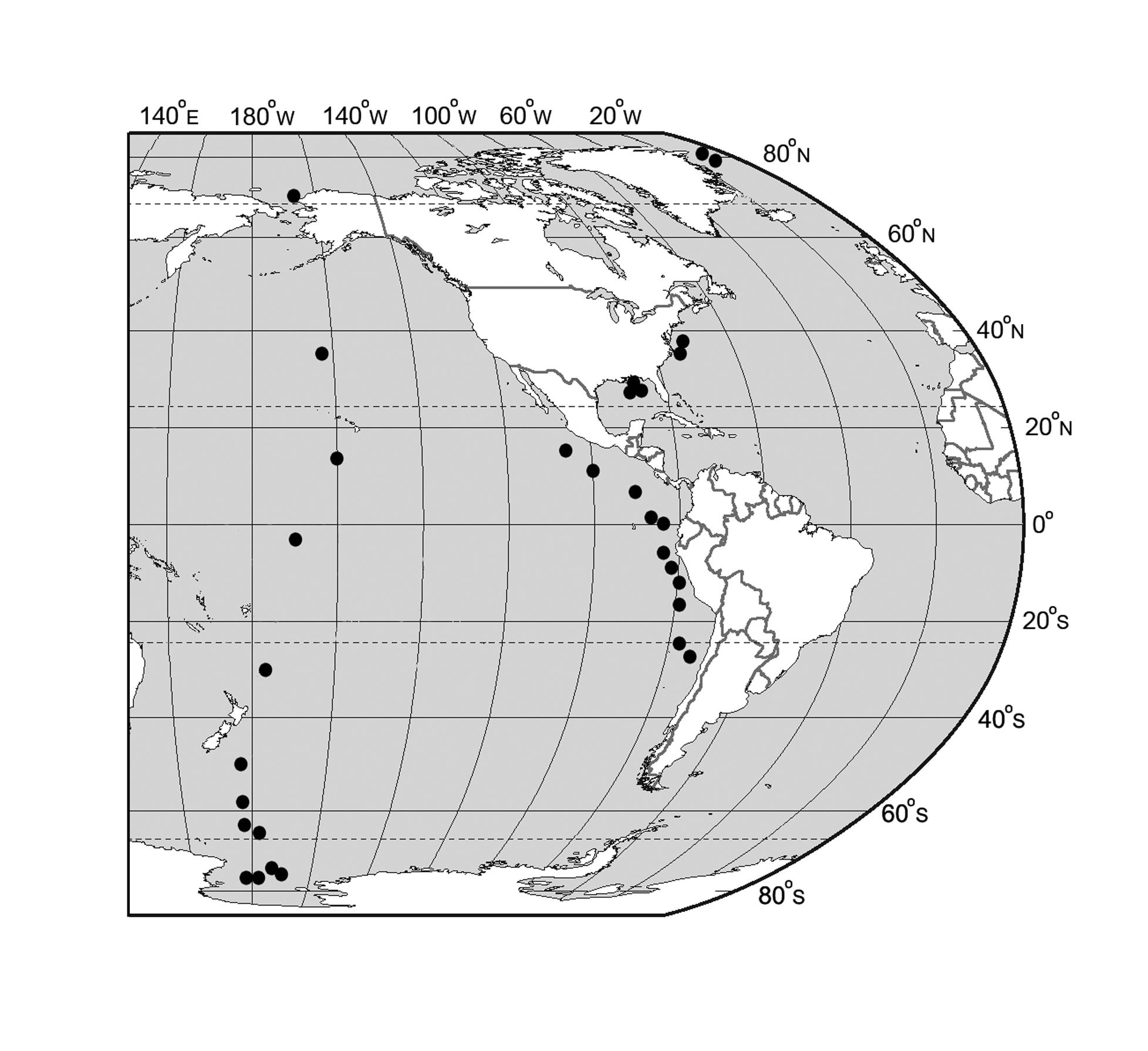

Supplement: Figure S1 — Map of sampling locations. (TIFF) [file pone.0028900.s001.tif]
