# Supplementary material for: Latitudinal Gradients in Degradation of Marine Dissolved Organic Carbon
Source: PLoS One. 2011 Dec 28;6(12):e28900. doi: 10.1371/journal.pone.0028900 (PMC3247214; doi:10.1371/journal.pone.0028900)
Supplement: Table S1 — Sampling dates and rates of enzymatic hydrolysis (nmol monomer L−1 h−1) of all substrates at all stations, including standard deviations of triplicate incubations. (DOC) [file pone.0028900.s002.doc]

**SUPPLEMENTARY INFORMATION**

**Latitudinal gradients in degradation of marine dissolved organic carbon**

C. Arnosti, A.D. Steen, K. Ziervogel, S. Ghobrial, W.H. Jeffrey

| Station | Pullulan | Laminarin | Xylan | Fucoidan | Arabinogalactan | Chondroitin | Sampling date |
| --- | --- | --- | --- | --- | --- | --- | --- |
| J^a^ | 0 | 1.2 ± 0.3 | 1.7 ± 0.7 | 0 | 0 | 3.4 ± 0 | July 1999 |
| J^b^ | 0 | 1.4 ± 0.4 | 4.9 ± 0.5 | 2.1 ± 0.3 | 0.20 ± 0.13 | 8.0 ± 0.3 | Sept. 2001 |
| J^c^ | 0 | 2.4 ± 0.5 | 4.5 ± 0.7 | 1.5 ± 0.3 | 0 | 8.9 ± 0.9 | Aug. 2007 |
| J | 0 | 0.25 ± 0.05 | 0.90 ±0.04 | 1.6 ± 0.3 | 0 | 5.0 ± 0.2 | Aug. 2008 |
| AB^a^ | 0 | 2.7 ± 0.5 | 2.8 ± 0.4 | 0 | 0 | 2.9 ± 0.2 | June 2000 |
| P2 | 0 | 2.0 ± 0.2 | 4.5 ± 0.2 | 0 | 1.2 ± 0.3 | 16.8 ± 0.4 | Aug. 2003 |
| DO^d^ | 1.3 ± 0.6 | 11.6 ± 1.5 | 5.4 ± 0.2 | 4.5 ± 6.7 | 3.2 ± 2.7 | 20.9 ± 2.9 | Dec. 2006 |
| CO^e^ | 0.12 ±0.02 | 7.7 ± 0.41 | 13.3 ± 2.4 | 7.2 ± 0.8 | 8.0 ± 3.6 | 1.9 ± 0.1 | Sept. 2003 |
| P10 | 1.2 ± 0.4 | 10.6 ± 0.4 | 2.9 ± 0.4 | 0 | 0 | 3.0 ± 0.9 | Sept. 2003 |
| GOM1 | 4.5 ± 2.4 | 19.0 ± 1.1 | 19.1 ± 1.6 | 7.8 ± 0.2 | 8.2 ± 1.0 | 11.4 ± 0.2 | June 2001 |
| GOM11 | 3.8 ± 1.1 | 14.9 ± 0.3 | 21.6 ± 0.2 | 6.3 ± 0.8 | 5.8 ± 0.6 | 11.4 ± 1.1 | June 2001 |
| GOM072^f^ | 6.8 ± 0.1 | 16.6. ± 0.7 | 14.8 ± 0.5 | 5.3 ± 0.7 | 2.8 ± 0.4 | 37.3 ± 0.4 | Sept. 2007 |
| GOM073^f^ | 1.3 ± 0.4 | 16.4 ± 0.4 | 16.0 ± 0.2 | 7.7 ± 0.7 | 5.2 ± 0.1 | 22.1 ± 0.5 | Sept. 2007 |
| P15 | 3.8 ± 0.5 | 10.0 ± 1.1 | 6.1 ± 1.0 | 1.4 ± 0.2 | 1.4 ± 0.7 | 1.5 ± 0.5 | Sept. 2003 |
| BOT12 | 5.3 ± 0.5 | 20.9 ± 0.6 | 9.9 * | 0 | 0 | 7.6 ± 5.4 | Oct. 2002 |
| BOT10 | 28.4 ± 3.2 | 20.1 ± 0.3 | 2.3 ± 1.0 | 5.5 ± 0.8 | 0 | 9.3 ± 1.2 | Oct. 2002 |
| BOT8 | 2.9 * | 20.7 ± 1.2 | 6.9 ± 0.3 | 0 | 0 | 9.0 ± 0.3 | Oct. 2002 |
| T33^a^ | 1.5 ± 0.4 | 14.6 ± 0.5 | 17.0 ± 4.4 | 0 | 0.91 ± 0.2 | 3.4 ± 1.2 | Aug 2000 |
| BOT7 | 0 | 21.3 ± 0.3 | 0 | 0 | 0 | 5.1 ± 3.3 | Oct. 2002 |
| P21 | 3.9 ± 0.2 | 11.9 ± 0.6 | 12.4 ± 0.8 | 1.5 ± 0.4 | 1.1 ± 0.3 | 3.6 ± 2.3 | Sept. 2003 |
| BOT5 | 1.7 ± 0.6 | 12.9 ± 0.6 | 2.9 ± 2.3 | 0 | 0 | 0.69 ± 1.2 | Oct. 2002 |
| BOT4 | 0.68 ± 0.32 | 11.4 ± 1.2 | 8.1 ±0.7 | 0 | 1.6 ± 0.2 | 0.86 ± 0.4 | Oct. 2002 |
| BOT3 | 0.81 ± 0.3 | 8.7 ± 0.4 | 3.3 ± 0.7 | 0 | 0 | 2.4 ± 0.8 | Oct. 2002 |
| T15^a^ | 2.1 ± 0.9 | 14.2 ± 0.2 | 6.5 ± 3.1 | 0 | 2.5 ± 1.0 | 0 | July 2000 |
| BOT1 | 1.6 ± 1.3 | 5.3 ±0.7 | 0.44 ±0.09 | 0 | 0.13 ± 0.1 | 0 | Oct. 2002 |
| P27 | 2.1 ± 0.5 | 5.1 ±0.8 | 14.2 ± 0.2 | 1.5 ± 0.8 | 0 | 1.3 ± 0.8 | Sept. 2003 |
| T3^a^ | 0 | 5.7 ± 0.1 | 0 | 0 | 0 | 4.3 ± 1.9 | July 2000 |
| G1 | 0.79 ±0.45 | 2.1 ± 0.2 | 3.3 ±2.5 | 0 | 0 | No data | Dec. 2004 |
| R1 | 0 | 1.2 ± 0.3 | 0 | 0 | 0 | 1.5 ± 1.2 | Oct. 2005 |
| R3 | 0 | 1 ± 0.3 | 0 | 0 | 0 | 0 | Nov. 2005 |
| M9 | 0 | 0.16 ±0.07 | 0.29 ±0.02 | 0 | 0.36 ± 0.21 | 1.3 ± 0.05 | Nov. 2003 |
| G11B | 0 | 2.9 ±0.6 | 0 | 0 | 0 | 0.12 ± 0.11 | Jan. 2005 |
| R10C | 0 | 1.2 ± 0.3 | 0 | 0 | 0 | 0 | Nov. 2005 |
| G9A | 0 | 1.1 ± 0.2 | 0.97 ±0.95 | 0 | 0 | 0 | Dec. 2004 |
| R13f | 0 | 1.2 ± 0.3 | 0 | 0 | 0 | 0 | Nov. 2005 |

* single incubation (no replicates)

^a^ Data from Stn J in 1999, Stn. AB, Stn. T3, T15, and T33 from [23]

^b^ Data from Stn J in 2001 from [29]

^c^ Data from Stn J in 2007 from [53]

^d^ Data from [54]

^e^ Data from [55]

^f^ Data from [56]

**Additional References**

54. Ziervogel K, Arnosti C (2009) Enzyme activities in the Delaware Estuary affected by elevated suspended sediment load. Est Coast Shelf Sci 84: 253-258.

55. Steen AD, Hamdan L, Arnosti C (2008) Dynamics of high molecular weight dissolved organic carbon in the Chesapeake Bay: Insights from enzyme activities, carbohydrate concentrations, and microbial metabolism. Limnol Oceanogr 53: 936-947.

56. Steen AD, Ziervogel K, Ghobrial S., Arnosti C (in prep.) Functional variation among polysaccharide-hydrolyzing microbial communities in the Gulf of Mexico.
